# Chicago Veterinary College

**Published:** 1890-05

**Authors:** 


*The Chicago Veterinary College.*—The commencement exercises of the Chicago Veterinary College took place on March 20th, 1890, at the Madison Street Theatre, Chicago. The attendance was large.

The class officers were: President, J. F. Huston; first vice-president, J. F. Roe; second vice-president, L. D. Browne; secretary, Clarence Mills; treasurer, J. W. Connaway.

The Committee of Arrangements consisted of Messrs. J. A. Brown, Charles W. Heitzmann, G. G. Grundy, J. W. Parkinson, M. Y. Shaffer, A. H. Schussler, M. W. Stark.

The faculty of the college were present, consisting of A. H. Baker, V.S., professor of theory and practice of veterinary medicine, pathology of horse, ox, sheep, swine and dog; R. J. Withers, M.D., V. S., professor of obstetrics; Joseph Hughes, M.R.C.V.S., professor of veterinary anatomy, special and comparative; E. M. Reading, M.D., professor of physiology and histology; Finley Ellingwood, M.D., professor of chemistry; J. F. Ryan, V.S., professor of lameness, diseases of the feet and limbs, practical and pathological shoeing; F. S. Billings, professor of micro-histology and gross pathology; Percy Clark, M.D., assistant demonstrator of practical chemistry; Jonathan Periam, professor of hygiene, breeding, and general management of domestic animals; C. E. Sayre, D.V.S., professor of dental surgery and helminthology; A. H. Baker, V. S., professor of principles and practice of veterinary surgery; R. J. Withers, M.D.V.S., professor of materia medica and toxicology.

In the graduating class of fifty gentlemen, eighteen graduated in honor. Of these Dr. J. H. Houston had the highest honors; Dr. J. F. Roe secured the prize in anatomy and also in materia medica; Dr. T. A. Shipley obtained the prize in pathology. The names and addresses of the gentlemen who were graduated are:

Walter Allen, Dunlap, Ill.; Frederick W. Ashe, Brooklyn, N. Y.; Hugh Barnes, Brimfield, Ill.; Eugene F. Beckley, Rockford, Ill.; Major G. Benton, Coldwater, Mich; Levi E. Booth, Corydon, Iowa; John A. Brown, Streator, Ill.; Lorenzo D. Browne, Donovan, Ill.; Frank E. Burnham, Minneapolis, Minn.; Edwin A. Buxton, Stockbridge, Wis.; Fredrick J. Burley, Galveston, Texas; Joel E. Cloud, Spiceland, Indiana; John W. Connaway, Columbia, Mo.; George L. Crocker, Maroa, Ill.; Douglas S. Defenbaugh, Streator, Ill.; Ira Dilley, Roseville, Ill.; Joseph Donovan, Marysville, Kan.; Charles T. Eckles, Eyota, Minn.; Harrison H. George, Napoleon, Ohio; Theodore T. Green, Walworth, Wis.; George G. Grundy, Morrisonville, Ill.; Thomas J. Gunning, Neponset, Ill.; Charles W. Heitzmann, Summit, Miss.; Isaac F. Houston, Bathgate, N. D.; John W. Kaull, Frankport, Dak.; Elmer H. Kinnett, Chapin, Ill.; John W. Lefever, Warsaw, Ind.; Willard D. Linn, Kings, Ill.; Owen J McGurty, Charleston, Ill.; Christian A. Miller, Louisville, Ky.; Clarence Mills, Mt. Palatine, Ill.; John H. Miller, Malden, Ill.; George T. Netherton, Jameson, Mo.; James P. Norton, Norwalk, Ohio; Daniel O'Neill, Minneapolis, Minn.; James W. Parkinson, Wenona, Ill.; William C. Rayen, Nashville, Tenn.; John F Roe, Wabash, Ind.; Joseph F. Roub, Monroe, Wis.; Fred B. Rowan, Belvidere, Wis.; Francis S. Schoenleber, Chicago, Ill.; Arthur H. Schussler, Orland, Ill.; Milton Y. Shaffer, Carthage, Ind.; Trajan A. Shipley, Ada, Ohio; Marcus W. Stark, East Lemon, Pa.; Francis E. Stone, Burlington, Wis.; Nathan I. Stringer, Fairbury, Ill.; John C. Tasche, Sheboygan, Wis.; William A. Waite, Racine, Wis.; Nathaniel P. Whitmore, Mazon, Ill.

The junior class of 1889 and 1890 was a large one, and as a rule the examinations for the first year's course were most satisfactory.

